# Solvent-Free procedure of an A9 Peptide Dimer Exhibiting Specific HER2 Receptor Binding: Fluorescence Spectroscopy Evaluation of the Enhanced Binding Affinity

**DOI:** 10.1021/acs.jmedchem.5c01194

**Published:** 2025-07-24

**Authors:** Valentina Verdoliva, Giuseppe Digilio, Emanuela Iaccarino, Stefania De Luca

**Affiliations:** 1 Institute of Crystallography, National Research Council (CNR), Via Vivaldi, 43, Caserta 81100, Italy; 2 Department of Science and Technological Innovation, Università del Piemonte Orientale “A. Avogadro”, Alessandria 15121, Italy; 3 Institute of Biostructures and Bioimaging, National Research Council (CNR), Naples 80131, Italy

## Abstract

HER2-expressing cancers currently benefit from targeted therapies, including monoclonal antibodies and antibody-drug conjugates that specifically bind to the extracellular domain of the receptor. Peptides targeting HER2 represent promising candidates for the development of alternative molecular drugs. In this study, we report a dimeric version of the previously validated A9 peptide as a ligand specifically targeting HER2. The novel A9-PEG-A9 conjugate consists of two A9 peptides whose N-terminal amino groups are linked via a polyethylene glycol chain. It was synthesized using a solvent-free protocol and validated as an improved ligand, demonstrating enhanced water solubility and increased affinity for the model receptor HER2-DIVMP, as determined by the fluorescence spectroscopy titration method.

## Graphical abstract

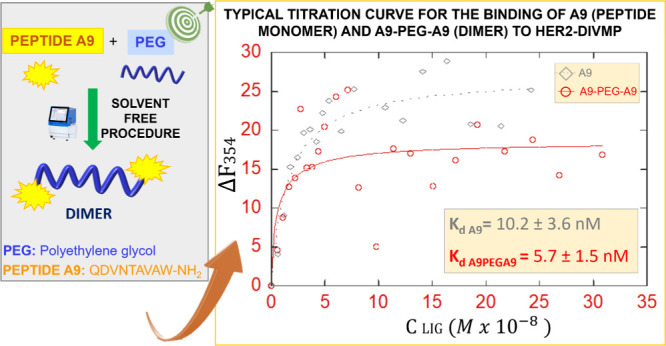

## Introduction

The overexpression or gene amplification of the HER2 receptor, a member of the ErbB receptor family, is associated with an aggressive breast cancer phenotype and has predictive value for other tumors, including lung, ovarian, colon adenocarcinomas, and salivary gland cancers.^1–3^ HER2 amplification leads to self-dimerization or dimerization with other ErbB receptors, resulting in continuous activation of HER signaling pathways.

Immunotherapy targeting the extracellular domain (EDC) of HER2 has been extensively developed as a specific approach to cancer research. Additionally, clinical therapies utilize specific antibodies or their fragments directly conjugated to anticancer drugs to deliver them to HER2-positive tumor cells.^4–9^

Several years ago, we developed the A9 peptide (QDVNTAVAW-NH_2_), a nine-residue linear peptide which binds specifically to the HER2 receptor with an affinity constant in the midnanomolar range.^10^ Such a small molecule holds great promise as an alternative to antibodies in HER2-targeted molecular imaging or therapeutic applications, but some improvements are needed. First, the HER2 binding affinity of the A9 peptide must be pushed further to approach that of the antibodies. This is crucial to exploiting A9 for molecular imaging diagnostic applications. Second, the hydrophobicity of A9 must be decreased, as it makes it difficult to handle the peptide in physiological solutions and contributes, to some extent, to nonspecific binding.

There are several examples in the literature about improved binding affinity and enhanced bioactivity of multivalent systems as compared to their monomeric analogs.^11^ We considered that making a dimer of the A9 peptide through a highly hydrophilic linker would enhance the HER2 binding affinity and improve the solubility of the A9 targeting peptide at the same time.

Herein, we report the procedure for conjugating two A9 peptide chains through a poly­(ethylene glycol) linker (PEG) under solvent-free conditions. PEGylation is a well-known process that enhances stability, prolongs circulation lifetime, and improves solubility without altering the peptide’s bioactive conformation.^12^ To assess the binding properties of the dimeric A9-PEG-A9 and make a comparison with those of the monomeric counterpart, we used a fluorescence spectroscopy assay developed previously. Such an assay is based on a small, soluble fragment of the HER2 receptor, called HER2-DIVMP model receptor.^13^ The measured dissociation constant of the dimeric A9-based ligand indicated an increased binding affinity compared to the monomeric analog.

## Results and Discussion

The conjugation process to bridge two A9 peptide chains by means of a PEG linker relied on a dicarboxy functionalized PEG derivative (molecular weight = 1000 Da), whose carboxyl groups were activated as N-hydroxysuccinimide (NHS) esters. This simple and versatile activation chemistry enables reaction with the primary N-terminal amine group of the peptide sequence. Such a coupling reaction is typically performed in a phosphate-buffered solution, and it is considered an effective and selective method. Since the A9 peptide is characterized by a poor solubility in water, both reagents were dissolved in a minimal amount of DMF. In fact, this solvent proved to be very effective in reaching a high reagent concentration in the reaction mixture. However, the mixture became increasingly thick until a very viscous phase was obtained after 3 h reaction time. Upon DMF removal and resuspension in water, the formation of a gel-like phase was observed, making further workup and product recovery impossible. We hypothesize that gel formation is associated with the incomplete removal of DMF, which likely facilitates the formation of supramolecular assemblies stabilized by peptide–peptide intermolecular interactions between peptide dimers.^14,15^

Therefore, we revised our synthetic strategy and implemented a solvent-free (SF) approach to efficiently obtain the desired A9 dimer.^16^ Nowadays, SF reactions are widely recognized as a milestone in organic synthesis, aligning with green chemistry principles that minimize environmental impact and reduce energy consumption. SF conditions address both of these objectives, particularly for industrial-scale chemical processes.

We explored an acylation reaction performed in the absence of solvents. Specifically, the N-terminus of peptide A9 was reacted with bifunctional PEG-Succinimidyl Carbonate, using only a catalytic amount of potassium carbonate as the peptide’s amino group itself acted as a base. The solid mixture was ground using a mortar and pestle, transferred into a vial, and irradiated with microwaves (MW) for 4 min at 80 °C (Scheme 1). The reaction was carried out by varying the reagent ratios to optimize the yield of the A9-PEG-A9 dimer with respect to the PEGylated monomer side product (Figure 1).

**Scheme 1:**
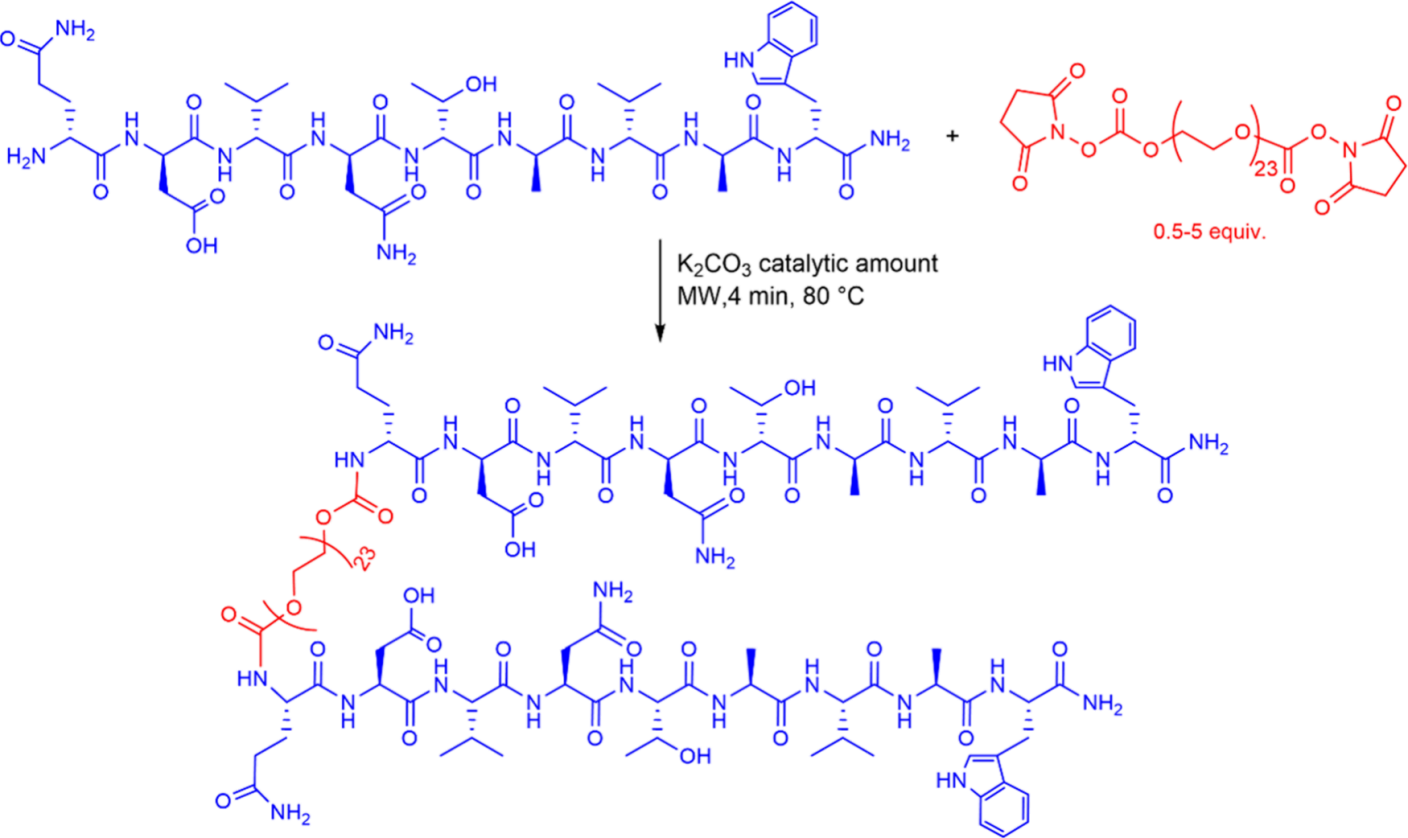
Solvent-Free Chemical Route to Prepare the Dimer A9-PEG-A9

**Figure 1:**
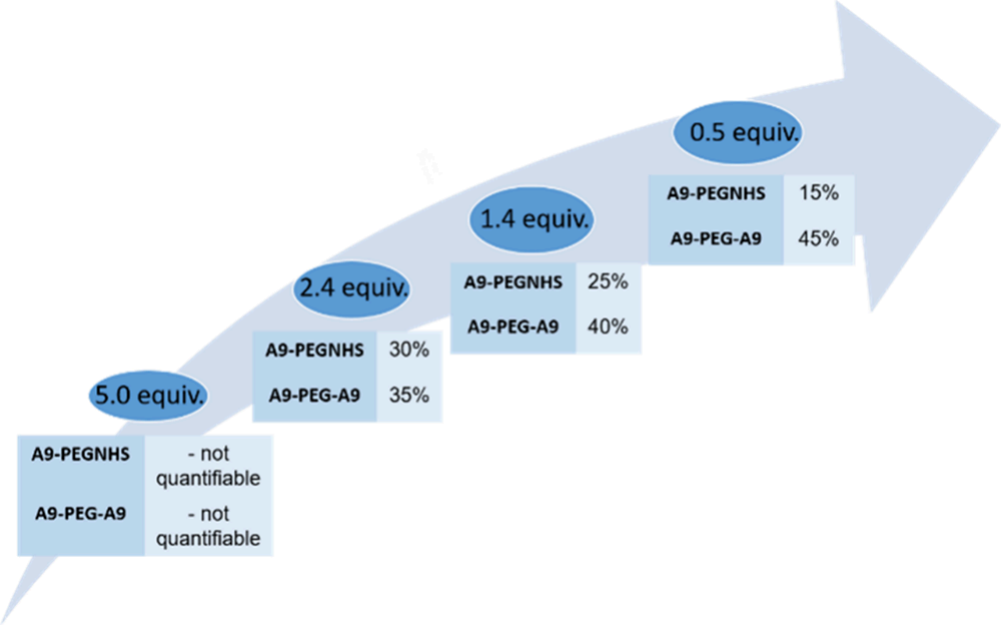
Reaction yield as a function of reagent mixing ratio (0.5–5 equiv).

As shown in Figure 2, comparing the HPLC peak areas of the monomer and dimer of the peptide-PEG conjugate, the optimal conditions involved using 0.5 equiv of NHS-PEG-NHS. A relatively small amount of PEG was necessary to drive the reaction toward dimer formation. In this regard, it is worth underlining that the equivalents of PEG derivative could not be furtherly lowered, since the mechanochemical process needs a minimum amount of reagents to be efficiently executed.

**Figure 2:**
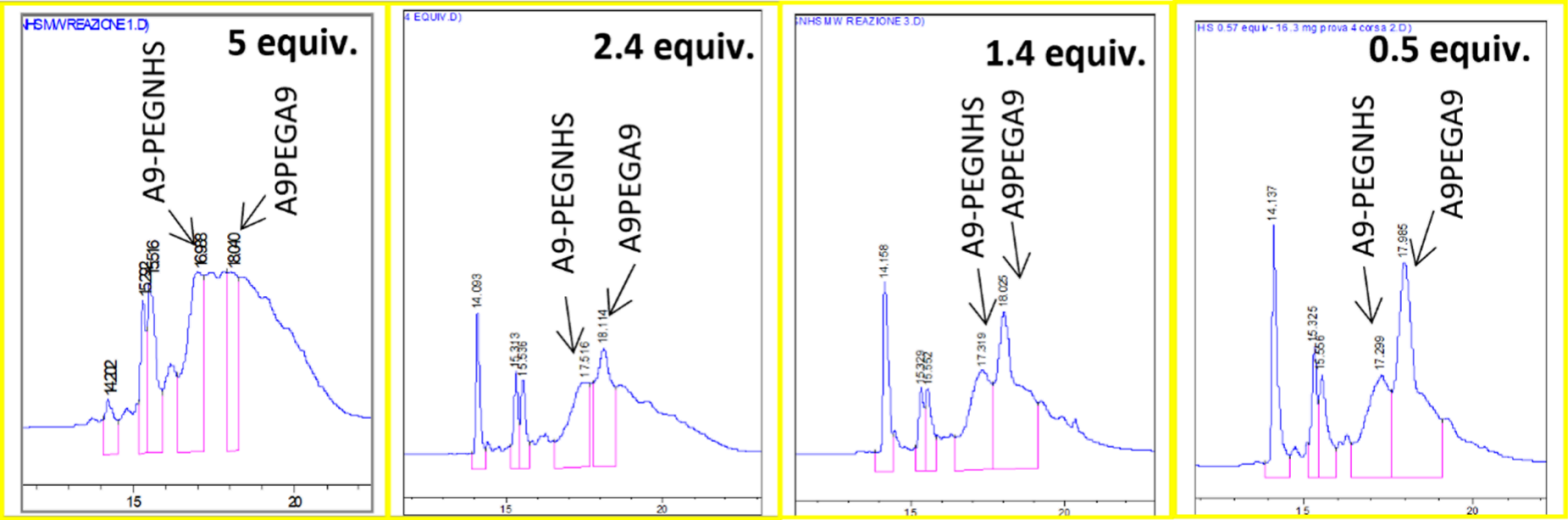
HPLC profiles of the reaction mixtures.

The reaction mixture (about 10 mg) was added to a small amount of water (3 mL) to dissolve the reaction products. The rapid formation of a clear solution of the reaction crude served as an initial indicator of the success of the synthetic protocol. The product was isolated by means of preparative HPLC and characterized by mass spectrometry (see Figure 3 and Supporting Information Figure S6). The product could be easily dissolved in water at a concentration of 3 mg mL^–1^. In our experience, A9 can be dissolved in water to a concentration <0.5 mg mL^–1^ after vigorous and prolonged stirring. This indicates that the dimerization of A9 by means of the PEG chain enhances the peptide water solubility.

**Figure 3:**
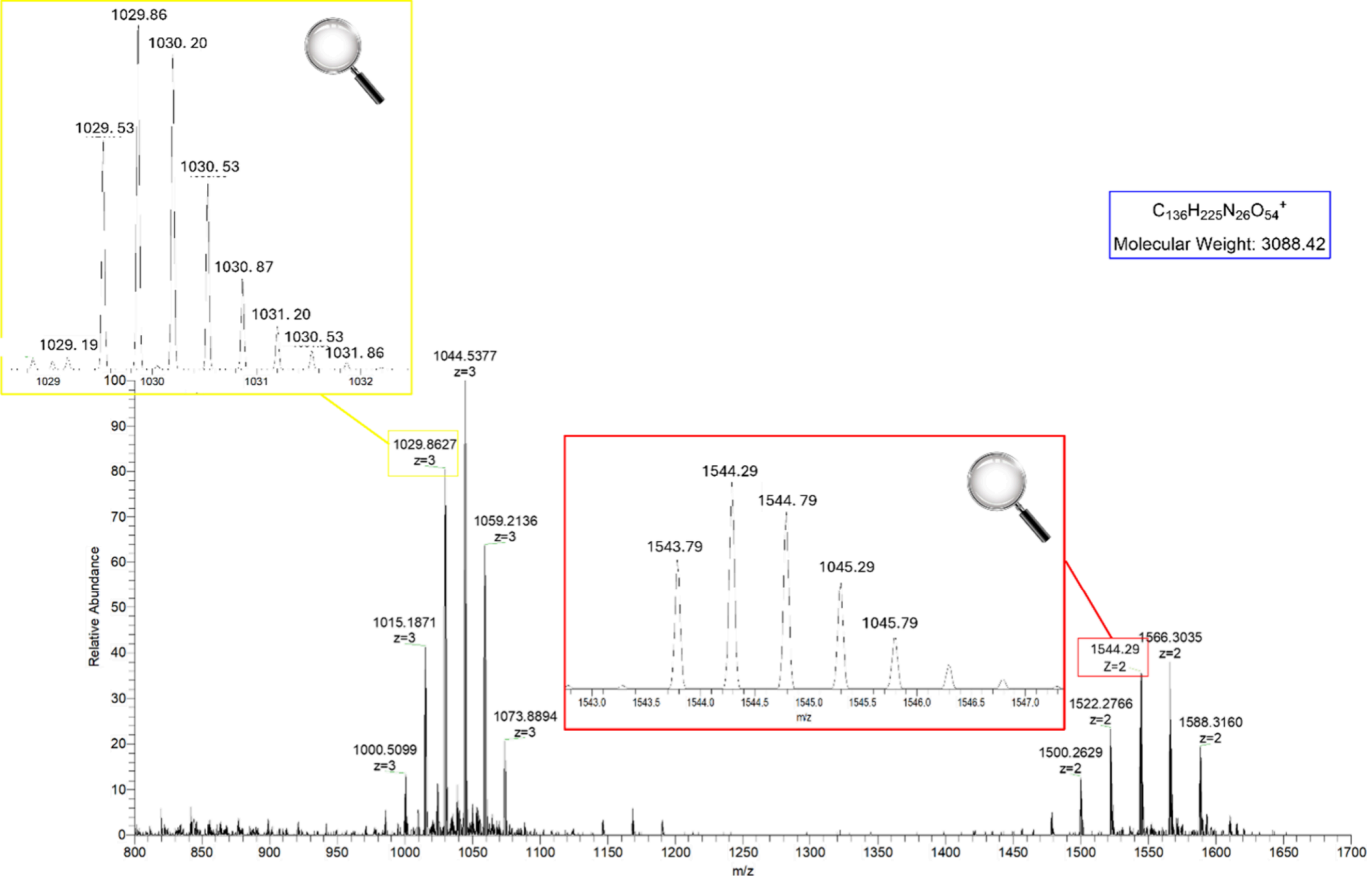
MS spectra of A9-PEG-A9 (dimer).

While environmental concerns are a primary driver for modernizing classical organic synthesis toward greener and more sustainable methodologies, our specific case demonstrated that eliminating solvents not only enhanced sustainability but also increased the yield and reduced reaction time. Specifically, the solvent-free protocol allowed us to overcome the issue of incomplete DMF removal, which hindered product recovery in our initial organic solution approach. By elimination of solvent cage effects, the solvent-free reaction significantly improved efficiency. Furthermore, to the best of our knowledge, our protocol represents the first example of a peptide being conjugated to a PEG linker in a solvent-free environment by homogenizing and mixing the reagents in the solid state under microwave activation. The efficiency of microwave ″heating″ has already been extensively discussed to justify the increased reaction rate.^17^ This acceleration is not due to thermal effects but rather to the polarizing electromagnetic field, which induces selective hotspot heating of individual reagents.

To confirm that the SF protocol yielded the expected fully soluble and functional A9-PEG-A9 dimer, several characterizations were conducted. Complete ^1^H NMR signal assignment of the purified A9-PEG-A9 dimer was achieved by 2D-NMR spectroscopy (2D-TOCSY, 2D-NOESY, and 2D-COSY). The ^1^H NMR spectra are consistent with the expected dimeric structure of the A9-PEG-A9 conjugate (Figure 4). The formation of the carbammic bonds between the PEG linker and the N-terminus Gln^1^ residue is confirmed by chemical shift comparison between the ^1^H NMR signals of the peptide in the monomeric (A9) form and in the dimeric (A9-PEG-A9) form, and by the detection in the 2D-NOESY spectrum of a NOE peak between the PEG methylene groups and Gln^1^ carbammic H_N_ (see the Supporting Information for a full ^1^H NMR assignment).

**Figure 4:**
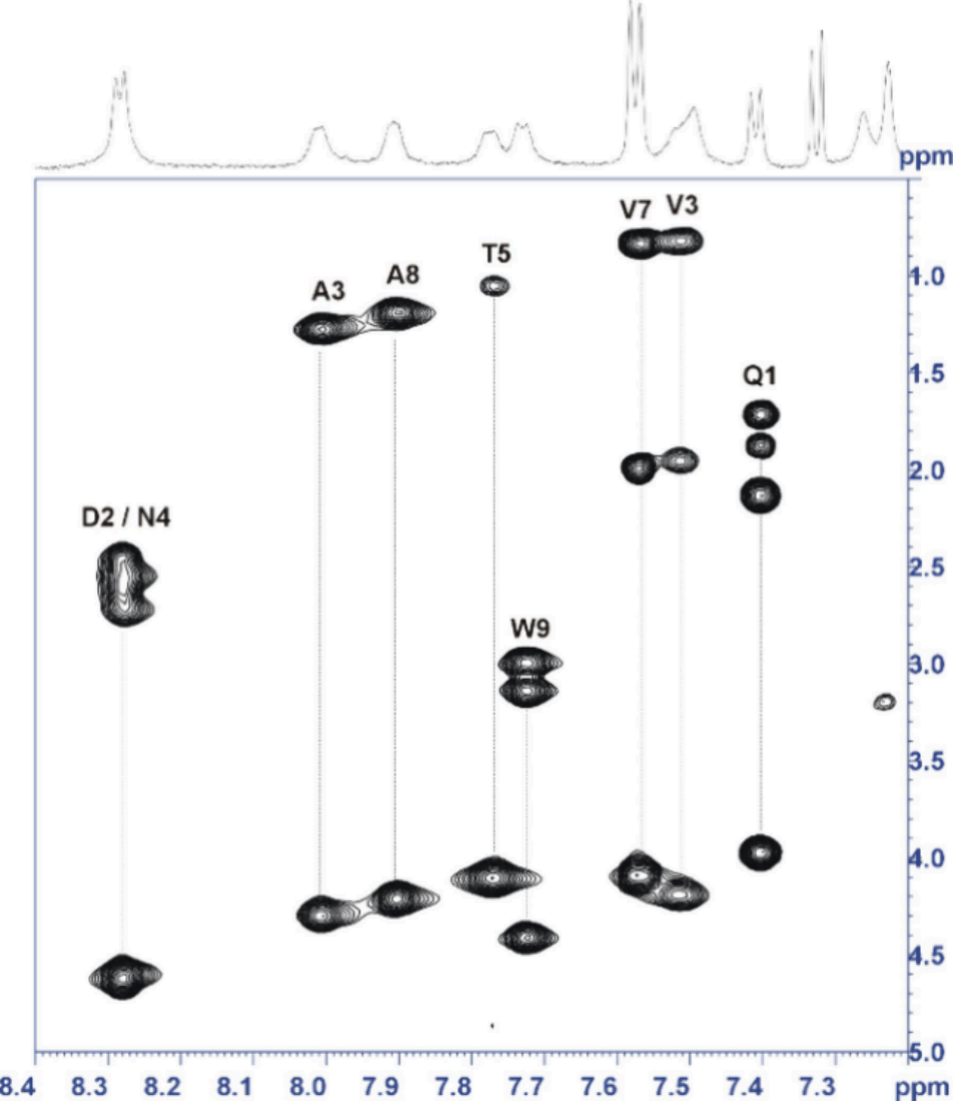
Expansion of the 2D-TOCSY NMR Spectrum of A9-PEG-A9 (DMSO-*d*_6_).

The fluorescence spectrum of the dimer, upon excitation at 280 nm, displayed a maximum around 354 nm, a wavelength close to that typical of free tryptophan in an aqueous solution (Figure 5, panel A). Finally, Circular dichroism showed that the A9-PEG-A9 dimer exhibits an unfolded conformation, with only a minimal content of secondary structure evidenced by the shoulder centered at 220 nm (Figure 5, panel B). Next, the stability of A9-PEG-A9 in human blood was assessed. After 8 h, only a small percentage of the dimer was lost, while after 24 h, the intact dimer accounted for more than 60% (Table 1).

**Figure 5:**
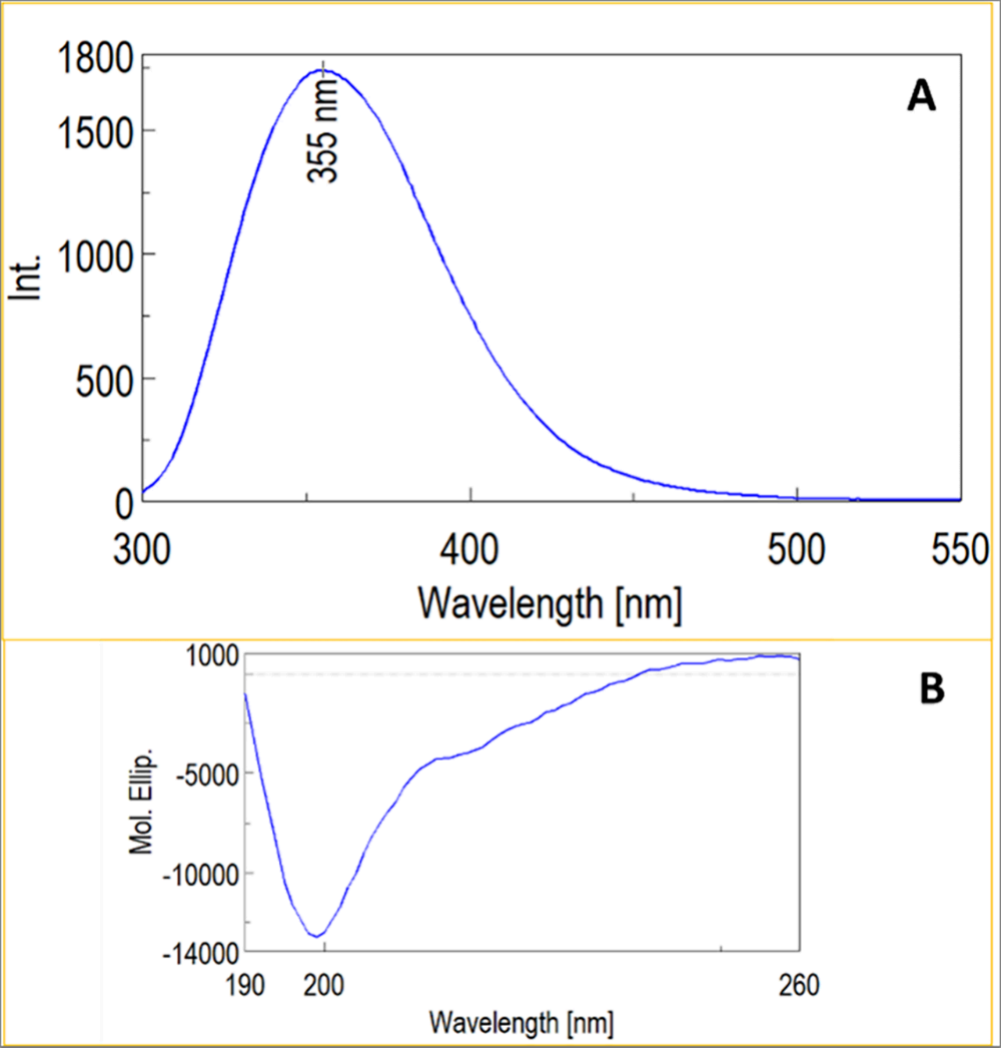
Emission fluorescence spectra of A9-PEG-A9 (dimer) upon excitation at 280 nm (panel A); circular dichroism (CD) spectra of A9-PEG-A9 (dimer) with representative secondary structure (panel B).

**Table 1:** Residual A9–PEG–A9 Peptide (%) in Human Serum at 37 °C

time (h)	residual A_9_–PEG–A_9_ (%)
0	100
1	85.39
4	82.47
8	83.42
24	63.96

Next, the binding affinity of the A9-PEG-A9 peptide ligand for its receptor model, HER2-DIVMP, was investigated by using the fluorescence spectroscopy titration method described previously (Figure [Fig fig7]6).^10^ Shortly, such a method involves an excitation wavelength of 280 nm, which excites simultaneously both tyrosyl and tryptophanyl fluorophores. Under these conditions, fluorescence resonance energy can be transferred from donor tyrosine residues to acceptor tryptophan residues. In the A9/HER2-DIVMP ligand–receptor bimolecular complex, the tyrosine Y_568_ residue belonging to the receptor fragment comes close enough to the tryptophan residue (W_35_) belonging to A9. Consequently, fluorescence resonance energy can be transferred from receptor Y_568_ to ligand W_35_ (in addition, fluorescence energy can be transferred from Y_568_ to W_592_ within the receptor), ultimately leading to an increase of tryptophan fluorescence emission (Figure 6).

**Figure 6:**
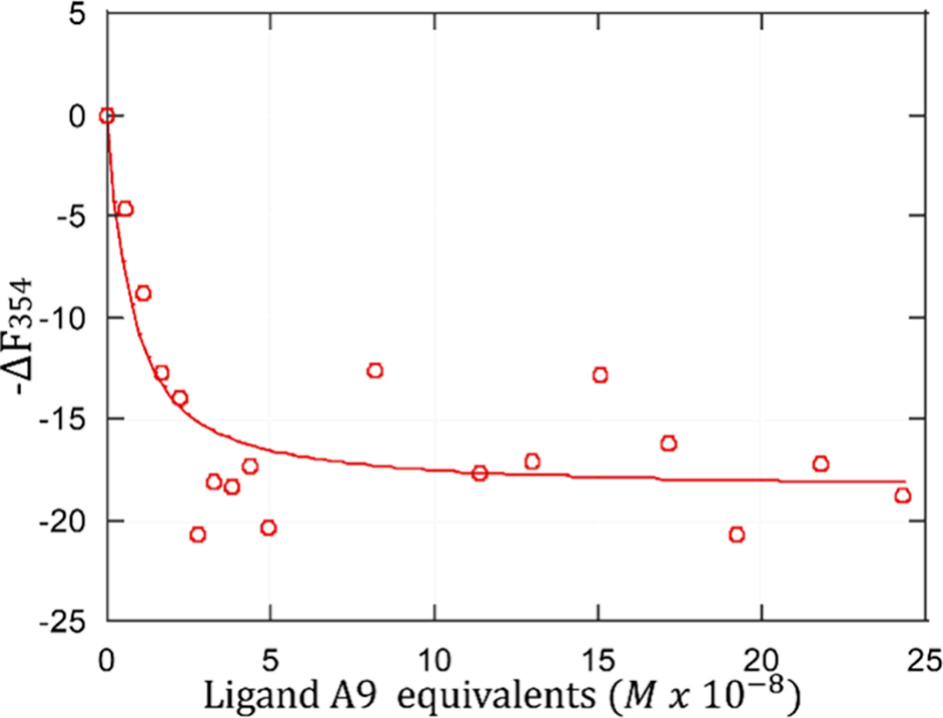
Typical titration curve for the binding of A9-PEG-A9 (dimer) to HER2-DIVMP (10 mM phosphate buffer, pH= 7.2). The receptor concentration was 0.0747 μM. The ordinate represents the residual fluorescence signal at 354 nm after the subtraction of the individual contributions of HER2-DIVMP and the peptide ligand.

According to our method, the receptor is titrated with the ligand while the tryptophan fluorescence emission is monitored at 354 nm. This fluorescence is corrected by subtracting (i) the fluorescence of the receptor in the absence of the ligand and (ii) that of the ligand at the same concentration in the absence of the receptor, yielding the fluorescence change (ΔF_354_). The resulting fluorescence change is plotted against the ligand concentration, producing a hyperbola-shaped binding isotherm. Computer-assisted fitting using a one-site binding model enables calculation of the dissociation constant, *K*_d_.^10^

Titrations with the A9-PEG-A9 dimer were carried out (λ_ex_ = 280 nm) at receptor concentrations ranging from 0.05 to 0.1 μM, consistently resulting in a fluorescence emission change at 354 nm, as expected. A representative ΔF_354_versus ligand concentration plot is shown in Figure 7. Note that in this plot, the ligand concentration is expressed in terms of equivalents of A9 peptide and not as equivalents of the A9-PEG-A9 dimer. The functional form and the sign of Δ*F*_354_ of the A9-PEG-A9 titration curve closely resemble those typical for the monomeric A9 peptide. This suggests that the dimeric and monomeric A9 forms share the same binding topology to the HER2-DIVMP receptor model. A one-site binding was hypothesized for both the A9 monomer and A9-PEG-A9 dimer. Computer-aided best-fitting of binding hyperbolas,^18^ based on the model for saturable specific binding (see the SI), yielded a dissociation constant (*K*_d_) of 5.7 ± 1.5 nM. This value is lower than that reported for A9 monomer (10.3 nM ± 3.6), indicating an increase of the binding affinity for the dimer.

**Figure 7:**
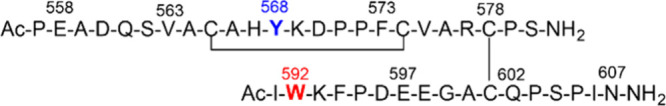
Chemical Structure of HER2-DIVMP.

## Experimental Section

### Materials

Fmoc protected amino acids, Rink Amide MBHA resin, *N*-hydroxybenzotriazole (HOBt), and benzotriazol-1-yl-oxy-tris-pyrrolidino-phosphonium (PyBOP) were purchased from Calbiochem-Novabiochem (Laufelfingen, Switzerland); piperidine and diisopropylethylamine (DIPEA) were purchased from Fluka (Milwaukee, WI); NHS-PEG-NHS 1k were purchased from Biopharma PEG; all solvents were purchased from Aldrich (St Louis, MI) or Fluka (Milwaukee, WI) and were used without further purification, unless otherwise stated.

### Synthetic Procedures and Characterization of A9-PEG-A9

#### Peptide Synthesis

C-Terminal amidated peptides were synthesized using a Rink Amide MBHA resin (0.74 mmol g^–1^ substitution; 50 μmol scale). In all syntheses, oxyma and DIC (oxyma/DIC) were employed as the standard activating reagents, following a specific protocol.^19^

Peptide synthesis was conducted using the solid-phase method on an SYRO I Biotage synthesizer, following the standard Fmoc-protecting group strategy. This process was fully automated and controlled by a computer-operated multiple peptide synthesizer, the Syro I from MultiSynTech GmbH (Witten, Germany). The instrument was equipped with a One U-Type Reactor Block featuring 24 positions and 5 mL reactors (PP-reactors, 5 mL with TF frit, code V050TF062, MultiSynTech GmbH, Witten, Germany). Additionally, the Syro I system included a vortex function capable of operating under variable conditions.

Amino acid coupling steps were monitored using the Kaiser test after 60 min coupling cycles. Fmoc deprotection was carried out with 20% piperidine in DMF for 5 + 10 min. The peptide N-terminus was acetylated by treating it with a mixture of acetic anhydride (4.7%) and pyridine (4%) in DMF for 10 min. Cleavage from the solid support and simultaneous deprotection of all side chains were performed by suspending the fully protected compound-resins in a TFA/H_2_O/TIS solution (97:2:1) for 3 h. Peptides were isolated by precipitation in cold diethyl ether and subsequently centrifuged to form a pellet.

For all RP-HPLC procedures, the solvent system used was H_2_O with 0.1% TFA (A) and CH_3_CN with 0.1% TFA (B), with detection carried out at 210 and 280 nm. Preparative RP-HPLC runs were performed using an HP Agilent Series 1200 apparatus equipped with a Phenomenex (Torrance, California) Gemini column (5 μm NX-C18 110 Å, 150 × 21.2 mm, AXIATM), with a flow rate of 15 mL min^–1^ and a linear gradient ranging from 5 to 70% B over 20 min.

LC-ESI-TOF-MS analyses were conducted using an Agilent 1290 Infinity LC system coupled with an Agilent 6230 TOF LC/MS System (Agilent Technologies, Cernusco sul Naviglio, Italy). The solvent system consisted of H_2_O with 0.05% TFA (A) and CH_3_CN with 0.05% TFA (B). Chromatographic separation was performed on a Phenomenex (Torrance, California) Jupiter column (3 μm C18 300 Å, 150 × 2.0 mm) with a linear gradient from 5% to 70% B over 20 min, and detection was carried out at 210 and 280 nm.

#### Solution-Phase Synthetic Procedure of NHS-PEG-A9-PEG-NHS (Strategy n.1)

A9 (20 mg) was dissolved in 2–3 mL of DMF in a balloon. After a few seconds, 0.25 equiv of NHS-PEG-NHS and 1 equiv of DIPEA (17 μL) were added. The reaction mixture was stirred at room temperature for 3 h and monitored by analytical RP-HPLC. The mixture became viscous and was not analyzed.

#### Solvent-Free Synthetic Procedure of NHSPEG-A9-PEGNHS (Strategy n.2)

A9 (10 mg) was reacted with 0.5–5 equiv of NHS-PEG-NHS in the presence of a catalytic amount of K_2_CO_3_. The mixture was manually milled by using an agate mortar. Then, it was transferred to a 0.5–2 mL microwave vial and irradiated at 80 °C for 4 min in a microwave oven (Biotage Initiator+, Sweden AB, Uppsala, Sweden). Subsequently, the solid mixture was dissolved in 3 mL of Milli-Q water.

The analytical method used to determine the purity of the compounds was LC-ESI-TOF-MS. Some other analyses were performed with an LC-MS system equipped with an Orbitrap high-resolution Q-Exactive Plus mass spectrometer (max resolution 280,000) equipped with an ESI ion source and connected to an Ultimate 3000 HPLC comprising a binary pump, an automated autosampler, and a multiwavelength diode array detector (hereafter Orbitrap ESI MS; ThermoFisher, Milano). These analytical methods confirmed that all compounds were ≥95% pure as assessed by HPLC.

A9-PEG-A9: [M + H]^+^ calculated 3088.42 *m*/*z*; [M + 2H]^+2^ calculated 1544.71, found 1544.2900; [M + 3H]^+3^ calculated 1030.1430, found 1029.8627.

#### HER2-DIVMP

One equiv portion of both peptides, chain A and chain B of HER2-DIVMP, was dissolved in ammonium bicarbonate aqueous solution (0.1 M; pH 7–8), until reaching the final concentration of 0.629 × 10–4 M. The reaction mixture was stirred at room temperature for 12 h and monitored by LC–MS analysis.

HER2-DIVMP: [M+ 4H]^+4^ calculated 1151.05 *m*/*z*; [M+ 4H]^+4^ found 1150.60 *m*/*z*.

#### UV–vis Spectrophotometry

The concentrations of all solutions used in fluorescence assays were determined by absorbance measurements using a Jasco V-730 Spectrophotometer_ETCS-761.

UV–vis spectra were recorded in the range of 250–600 nm at room temperature using 500 μL quartz cells, with blank correction. The experimental parameters were set as follows: scan speed of 200 nm/min, data interval of 0.2 nm, response time of 0.24 s, continuous scan mode, and a bandwidth of 1.0 nm.

#### Fluorescence Spectroscopy

Fluorescence spectra were recorded at room temperature on a Jasco Model ETC-115, equipped with a 1.0 cm quartz cell, with an excitation wavelength of 280 nm and an emission range of 300–550 nm. Equal excitation and emission bandwidths were used throughout experiments with an automatic recording speed and an automatic selection of the time constant.

The emission spectra of HER2-DIVMP alone (0.027 mM) and in complex with the peptide A9-PEG-A9 (at an equimolar ratio), both dissolved in a 10 mM phosphate buffer solution (pH 7.2), were recorded upon excitation at 280 nm.

In a typical experiment, the initial sample volume was 1 mL, containing HER2-DIVMP (0.0747 μM) in a 10 mM phosphate buffer solution (pH 7.2). Appropriate aliquots of a peptide A9-PEG-A9 stock solution (0.553 μM) prepared in the same buffer were added to reach a final concentration of 0.308 μM. At each titration step, the sample was mixed and fluorescence measurements were taken after allowing the receptor/ligand mixtures to equilibrate.

To estimate the fluorescence specifically arising from the interaction between A9-PEG-A9 and HER2-DIVMP, a blank titration series was performed by adding equal amounts of the peptide to a fixed volume of buffer solution. Final spectra were obtained after blank correction, adjustment for dilution, and subtraction of the separate fluorescence contributions of the peptide ligands and receptor fragment from the total fluorescence of the assay mixtures.

All titrations were performed in triplicate, monitoring the fluorescence intensity at 354 nm. The fluorescence signal at 354 nm was plotted against the peptide ligand concentration and fitted using a sigmoidal dose–response binding model in Prism 5 (GraphPad, La Jolla, CA) (Figure 7).

#### Circular Dichroism

CD spectra were recorded using 0.027 mM solutions of A9-PEG-A9 in 10 mM phosphate buffer, pH 7.2. The spectra were normalized to the mean residue ellipticity ([Y]) and the secondary structure content was estimated using the offline Jasco J-1500 CD spectrometer.

#### Nuclear Magnetic Resonance (^1^H NMR)

NMR spectra were acquired with a Bruker Avance spectrometer operating at 14 T (corresponding to a proton Larmor frequency of 600 MHz), equipped with an inverse Z-gradient 5 mm BBI probe. A9-PEG-A9 or A9 samples (about 1 mg each) were dissolved in dmso-*d*_6_ and spectra were acquired at 298 ± 0.1 K. 2D-TOCSY spectra were acquired with the Bruker mlevph pulse program in the States-TPPI phase-sensitive mode, with a 2.5 s relaxation delay, 32 scans, 16 dummy scans, 2048 × 400 data points, 17 ppm spectral width (both F2 and F1), and 100 ms mixing time. Data were treated with squared cosine window functions (both along F2 and F1) prior to complex FT. 2D-NOESY spectra were acquired with the Bruker noesyph pulse program in the States-TPPI phase-sensitive mode. Acquisition and processing parameters were as for 2D-TOCSY spectra but with a NOESY mixing time of 300 ms. Double quantum filtered 2D-COSY spectra were acquired with the Bruker cosydfph pulse program in the States-TPPI phase-sensitive mode (acquisition parameters are as above). Spectra were processed by using the Bruker Topspin 4.0.7 software package. Sequence-specific resonance assignment was carried out by the Computed Aided Resonance Assignment software package.^20^

#### Peptide Stability in Human Serum

The A9–PEG–A9 peptide was dissolved in 10% (v/v) human serum (Sigma-Aldrich, Milan, Italy) to obtain a final peptide concentration of 1 mg/mL. The solution was incubated at 37 °C for up to 24 h. At designated time points (1, 4, 8, and 24 h), 30 μL aliquots (corresponding to approximately 330 μg of peptide) were withdrawn and added to ice-cold ethanol to reach a final ethanol concentration of 90% (v/v). Samples were incubated on ice for 15 min and then centrifuged at 12,000 rpm for 5 min at 4 °C to eliminate the precipitated proteins. The collected supernatants were diluted with water 0.1% trifluoroacetic acid (TFA) to reach a final peptide concentration of 0.1 mg/mL. Peptide stability was assessed via reverse-phase high-performance liquid chromatography (RP-HPLC), using Jupiter 4 μm Proteo 90 Å, LC Column 250 mm × 10 mm, Ea. A linear gradient elution was applied, ranging from 1% to 85% solvent B over 15 min at a flow rate of 0.8 mL/min. Solvent A was water with 0.1% TFA, and solvent B was acetonitrile with 0.1% TFA.

The percentage of intact peptide remaining at each time point was estimated by the HPLC peak area of A9-PEG-A9 (Table 1).

## Conclusions

In conclusion, we have developed a solvent-free method for the synthesis of the A9-PEG-A9 dimer, which exhibits a slightly increased receptor binding affinity and significantly enhanced water solubility compared with the parent monomeric A9 peptide. This study demonstrates that the multimerization of bioactive small-peptide ligands using a short PEG-based linker is a viable strategy to enhance their potency. To the best of our knowledge, this work presents the first protocol that enables PEGylation and dimerization of a preformed bioactive peptide through microwave-assisted, solid-state mechanochemistry. Notably, no solvents or liquid reagents were required during the process.

## Supplementary Material





## Notes

HPLC profiles, MS spectra, NMR chemical shift, and spectra of peptide conjugates (PDF)
Molecular formula strings (CSV)

## Abbreviations

^1^H NMR: nuclear magnetic resonance
2D-COSY: two-dimensional correlation spectroscopy
2D-NOESY: two-dimensional nuclear Overhauser effect spectroscopy
2D-TOCSY: two-dimensional total correlation spectroscopy
A9: QDVNTAVAW-NH_2_
CD: circular dichroism
DIPEA: diisopropylethylamine
DIC: diisopropylcarbodiimide
DMF: dimethylformamide
EDC: extracellular domain
Gln: glutamine
HER2: human epidermal growth factor receptor 2
HOBt: *N*-hydroxybenzotriazole
HPLC: high-performance liquid chromatography
MW: microwaves
NHS: *N*-hydroxysuccinimide
PEG: polyethylene glycol linker
PyBOP: benzotriazol-1-yl-oxy-tris-pyrrolidinophosphonium hexafluorophosphate
SF: solvent-free
TFA: trifluoroacetic acid
TIS: triisopropylsilane
UV–vis: ultraviolet–visible
H_2_O: water
