# Supplementary material for: Solvent-Free procedure of an A9 Peptide Dimer Exhibiting Specific HER2 Receptor Binding: Fluorescence Spectroscopy Evaluation of the Enhanced Binding Affinity
Source: J Med Chem. 2025 Jul 24;68(15):16299–305. doi: 10.1021/acs.jmedchem.5c01194 (PMC12406181; doi:10.1021/acs.jmedchem.5c01194)
Supplement: Supplementary file 1 [file jm5c01194_si_001.pdf]

Supporting Information

Solvent-Free procedure of an A9 Peptide Dimer Exhibiting Specific HER2 Receptor Binding: Fluorescence Spectroscopy Evaluation of the Enhanced Binding Affinity

Valentina Verdoliva[1], Giuseppe Digilio[2], Emanuela Iaccarino[3], Stefania De Luca[3]*

1 Institute of Crystallography, National Research Council (CNR), Via Vivaldi, 43, 81100 Caserta, Italy

2 Department of Science and Technological Innovation, Università del Piemonte Orientale "A. Avogadro", 15121 Alessandria, Italy

3 Institute of Biostructures and Bioimaging, National Research Council (CNR), 80131 Naples, Italy

Corresponding author's email address: stefania.deluca@cnr.it

TABLE OF CONTENTS

- **Preparative HPLC of A9 performed with 0.5-5 equiv. of NHS-PEG-NHS……………..S2**
- **HPLC, TIC, MS spectra of dimer A9-PEG-A9…………………………………………S6**
- **^{1}H and ^{13}C NMR spectra of dimer A9-PEG-A9……………………………………...S7**
- **TIC spectra of A9–PEG–A9 degradation in Human Serum over 24 hours…………S15**
- **TIC and MS spectra of monomer A9-PEG-NHS…………………………………..…..S16**

Solution-phase Synthetic procedure of NHS-PEG-A9-PEG-NHS 1k

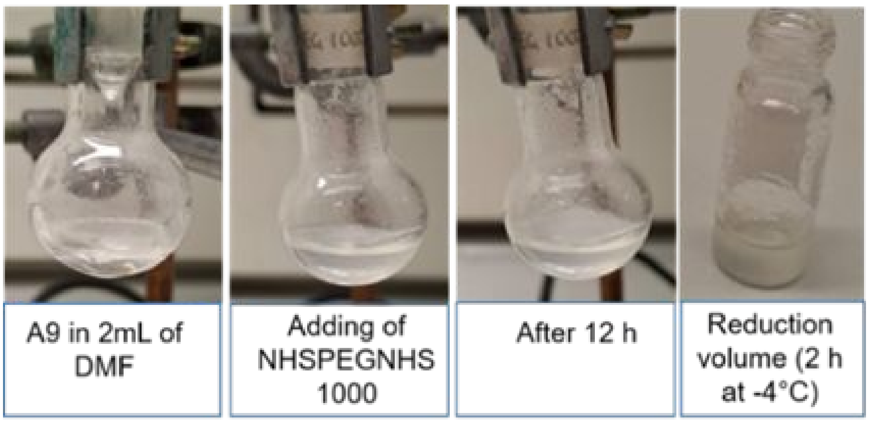

Figure 1S. Standard solution synthesis of NHSPEG-A9-PEGNHS 1000

Solvent free synthetic procedure of NHS-PEG-A9-PEG-NHS 1k

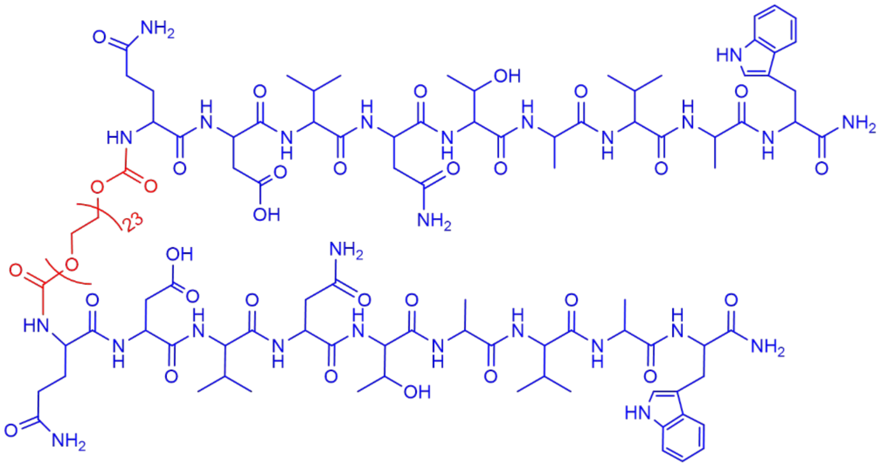

Figure 2S. Chemical structure of dimer A9-PEG-A9

Reaction of A9 performed with 5 equiv. of NHS-PEG-NHS

7.6 mg of A9 was reacted with 5 equiv. of NHS-PEG-NHS in the presence of a catalytic amount of K_2CO_3. The mixture was manually milled by using an agate mortar.

The mixture, placed in a 0.5–2 mL microwave vial, was irradiated for 4 min at 80 °C in a microwave oven (Biotage® Initiator+, Sweden AB, Uppsala, Sweden). Then the solid state mixture was dissolved in 3 mL of H_2O milli Q.

$TR_{A9\text{-}PEG\text{-}A9}$= 18.040 min; White solid

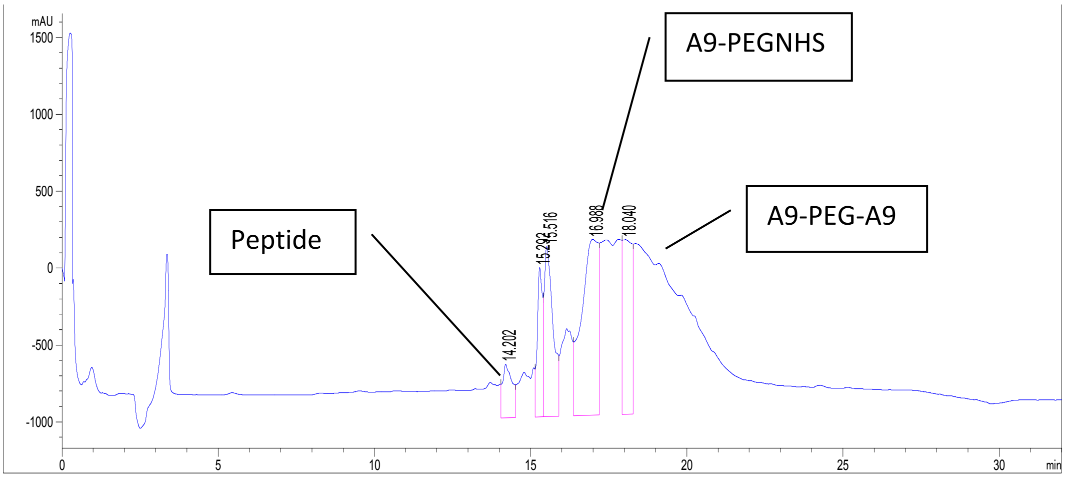

Figure 3S. HPLC profile of the reaction crude product

Reaction of A9 performed with 2.4 equiv. of NHS-PEG-NHS

15.5 mg A9 was reacted with 2.4 equiv. of NHS-PEG-NHS in the presence of a catalytic amount of K_2CO_3. The mixture was manually milled by using an agate mortar.

The mixture, placed in a 0.5–2 mL microwave vial, was irradiated for 4 min at 80 °C in a microwave oven (Biotage® Initiator+, Sweden AB, Uppsala, Sweden). Then the solid state mixture was dissolved in 3 mL of H_2O milli Q.

$TR_{A9\text{-}PEG\text{-}A9}$= 18.114 min; White solid

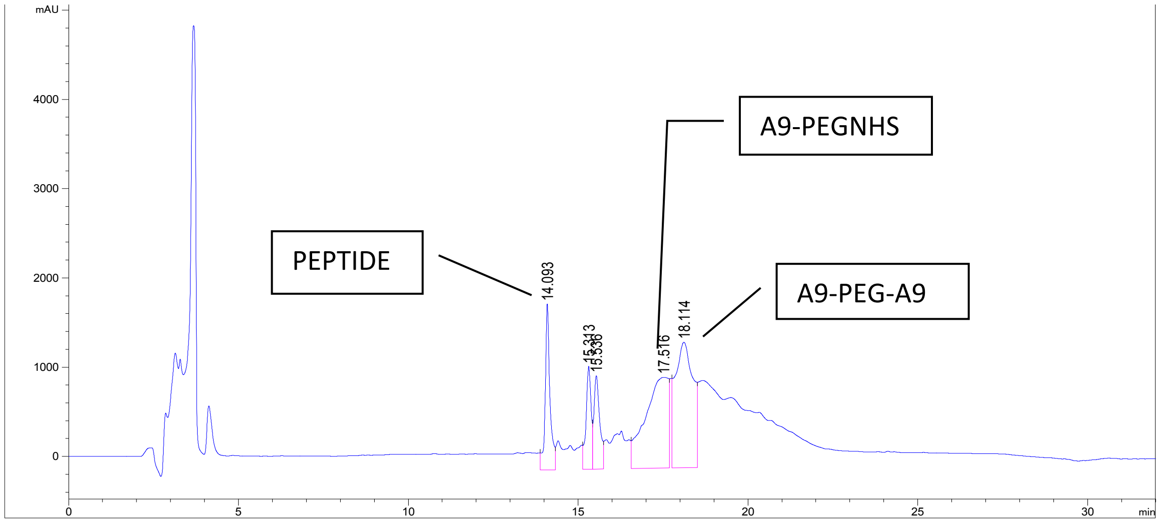

Figure 4S. HPLC profile of the reaction crude product

Reaction of A9 performed with 1.4 equiv. of NHS-PEG-NHS

15.7 mg A9 was reacted with 1.4 equiv. of NHS-PEG-NHS in the presence of a catalytic amount of K_2CO_3. The mixture was manually milled by using an agate mortar.

The mixture, placed in a 0.5–2 mL microwave vial, was irradiated for 4 min at 80 °C in a microwave oven (Biotage® Initiator+, Sweden AB, Uppsala, Sweden). Then the solid state mixture was dissolved in 3 mL of H_2O milli Q.

$TR_{A9\text{-}PEG\text{-}A9}$= 18.025 min; White solid

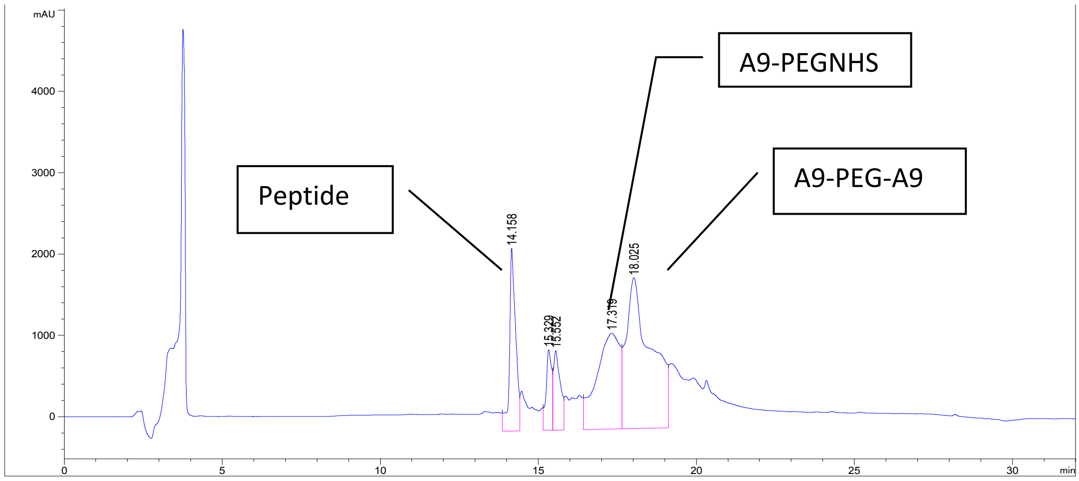

Figure 5S. HPLC profile of the reaction crude product

Reaction of A9 performed with 0.5 equiv. of NHS-PEG-NHS

20.7 mg A9 was reacted with 0.5 equiv. of NHS-PEG-NHS in the presence of a catalytic amount of K_2CO_3. The mixture was manually milled by using an agate mortar.

The mixture, placed in a 0.5–2 mL microwave vial, was irradiated for 4 min at 80 °C in a microwave oven (Biotage® Initiator+, Sweden AB, Uppsala, Sweden). Then the solid state mixture was dissolved in 3 mL of H_2O milli Q.

$TR_{\text{A9-PEG-A9}}$= 17.995 min; White solid

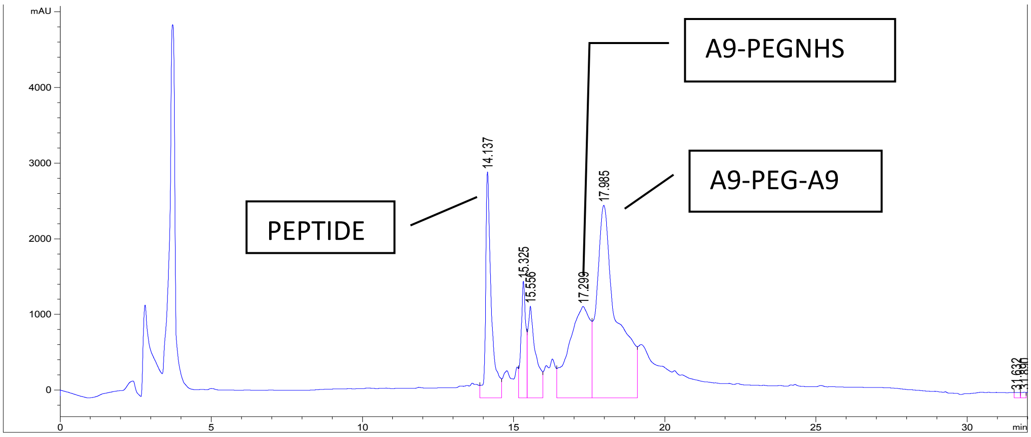

Figure 6S. HPLC profile of the reaction crude product

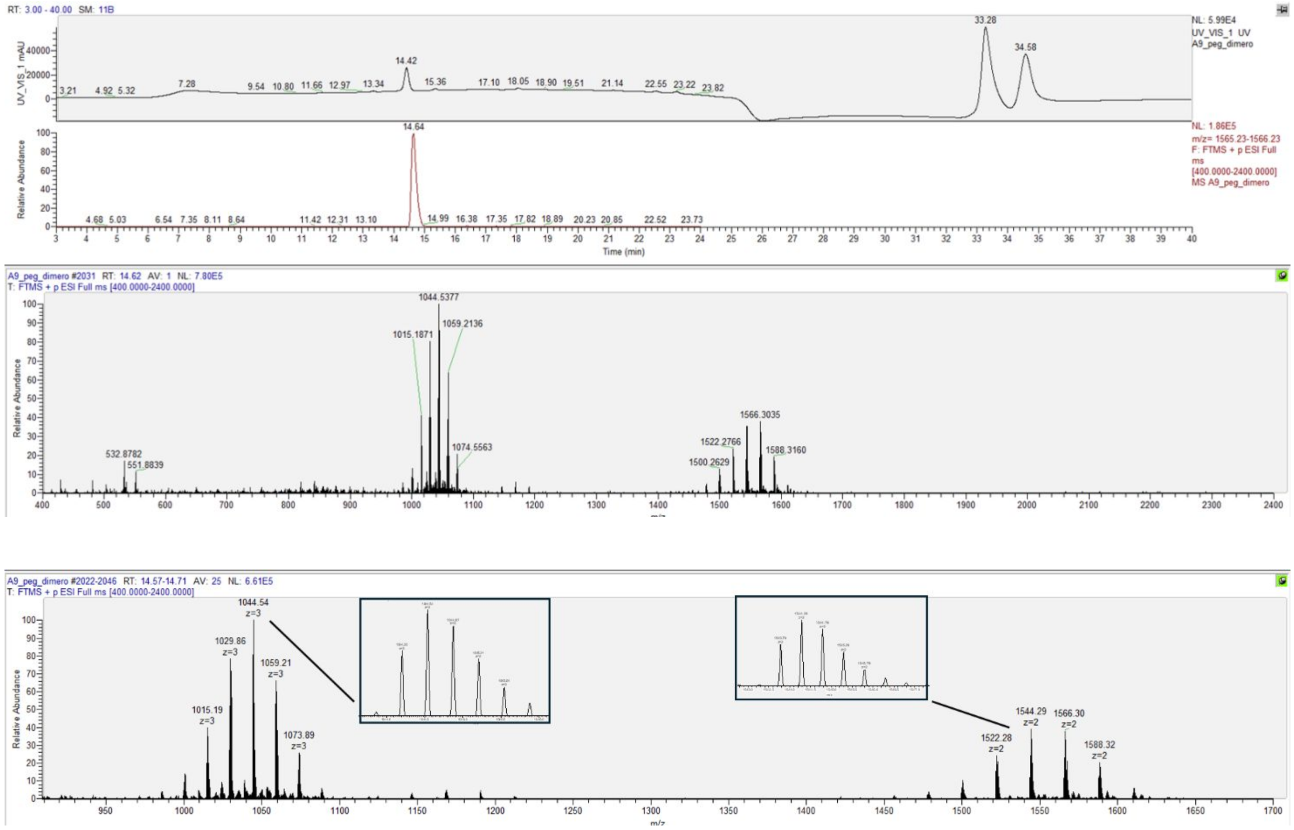

Figure 7S. HPLC, TIC and MS spectra of dimer A9-PEG-A9

^{1}H NMR spectra of dimer A9-PEG-A9

^{1}H-NMR δ, ppm (600 MHz, dmso-d_6, 298K): 10.78 (s, 1H, W^9 NH ring), 8.28 (overlapping doublets, 2H: N^4 H_N ; D^2 H_N), 8.01 (d, br, 1H, A^6 H_N), 7.91 (d, br, 1H, A^8 H_N), 7.77 (d, br, 1H, T^5 H_N), 7.73 (d, br, 1H, W^9 H_N), 7.57 (overlapping doublets, 2H: W^9 ring, V^7 H_N), 7.51 (d, br, overlap, 1H, V^3 H_N), 7.49 (s, br, overlap, 1H, N^4 $CONH_2$), 7.41 (d, 1H, Q^1 H_N), 7.33 (d, 1H, W^9 ring), 7.26 (s, br, 1H, C-term $CONH_2$), 7.23 (s, br, 1H, Q^1 $CONH_2$), 7.15 (s, br, 1H, W^9 ring), 7.06 (t, overlap, 1H, W^9 ring), 7.05 (s, br, overlap, 1H, C-term $CONH_2$), 7.01 (s, br, overlap, 1H, N^4 $CONH_2$), 6.98 (t, overlap, 1H, W^9 ring), 6.75 (s, br, 1H, Q^1 $CONH_2$), 4.87 (s, br, 1H, T5 -OH), 4.64 (m, br, 1H, N^4 Hα), 4.58 (m, 1H, D^2 Hα), 4.42 (m, br, 1H, W^9 Hα), 4.30 (m, br, 1H, A^6 Hα), 4.22-4.20 (overlapping multiplets, 2H: A^8 Hα; V^3 Hα), 4.09-4.07 (overlapping multiplets, 3H: V^7 Hα, T^5 Hα, T^5 Hβ), 3.98 (m, br, 1H, Q^1 Hα), 3.14 (m, br, 1H, W^9 Hβ1), 3.53 (s, 100H, PEG -CH_2O-), 2.99 (dd, 1H, W^9 Hβ2), 2.72 (m, br, 1H, D^2 Hβ1), 2.62 (overlap with dmso-d_6 ^{13}C satellite, N^4 Hβ1), 2.54 (overlap with dmso-d_6, D^2 Hβ2), 2.45 (overlap with dmso-d_6, N^4 Hβ2), 2.12 (m, 2H: Q^1 Hγ), 1.98-1.97 (overlapping multiplets, 2H: V^7 Hβ, V^3 Hβ), 1.87 (m, 1H, Q^1 Hβ1), 1.72 (m, 1H, Q^1 Hβ2), 1.28 (d, 3H, A^6 -CH_3), 1.19 (d, 3H, A^8 -CH_3), 1.05 (d, 3H, T^5 -CH_3), 0.83-0.81 (overlapping multiplets, 12H, V^7 and V^3 -CH_3).

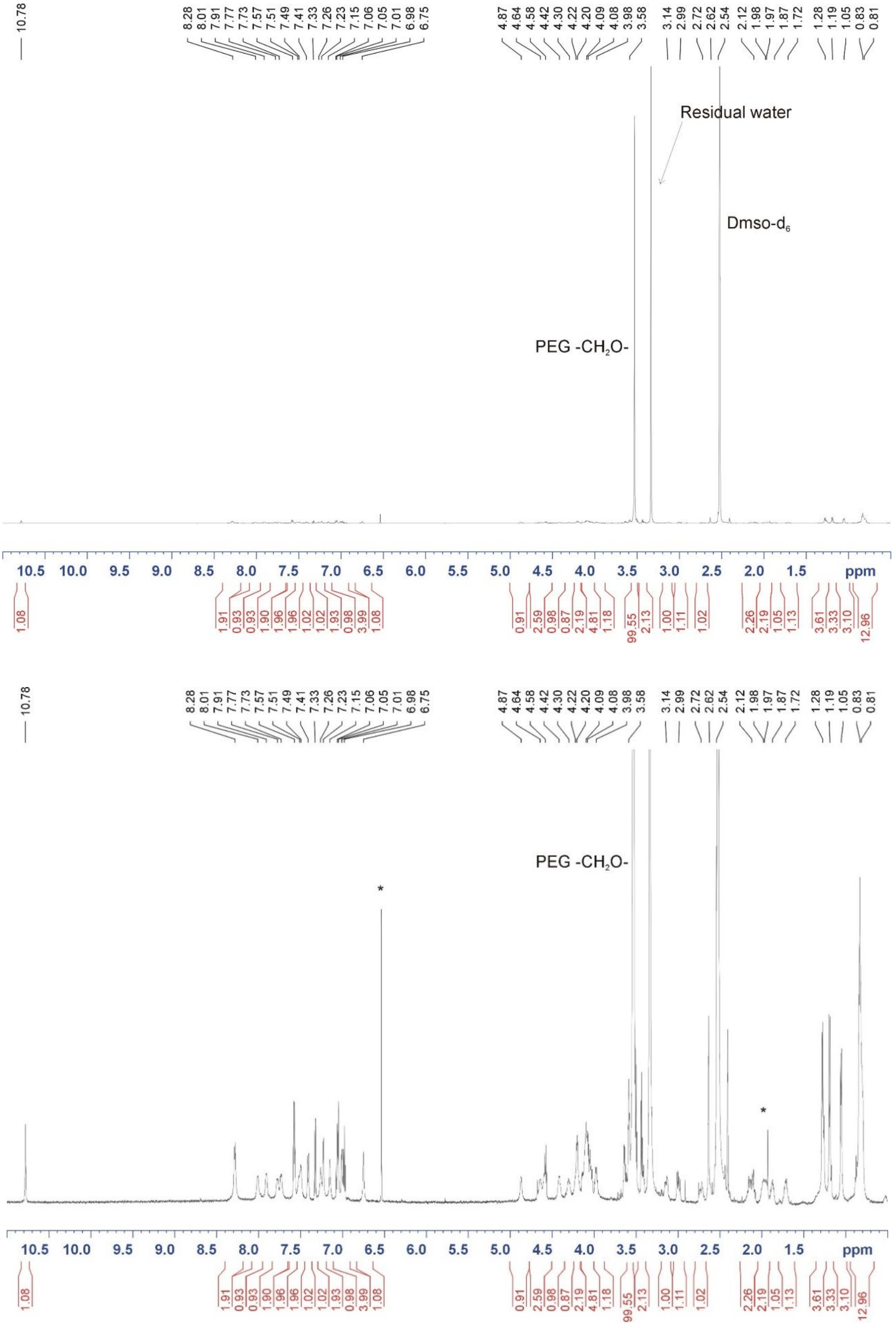

32x magnification (y-scale)

Figure 8S. ^{1}H-NMR (600 MHz, dmso-d_6, 298 K), with expanded regions (asterisks denote impurities).

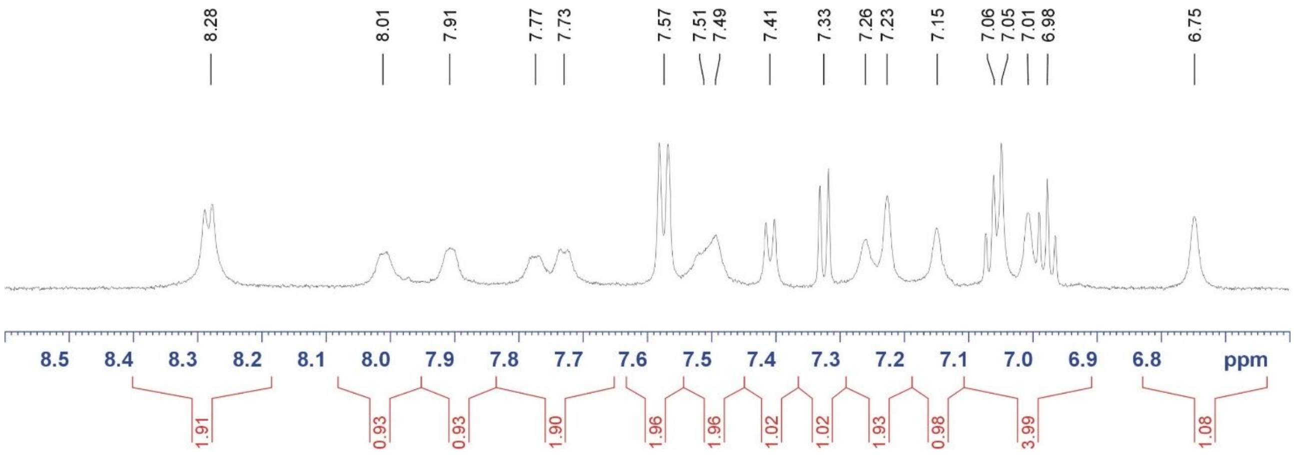

Figure 9S. Expansion of the 8.6-6.6 region

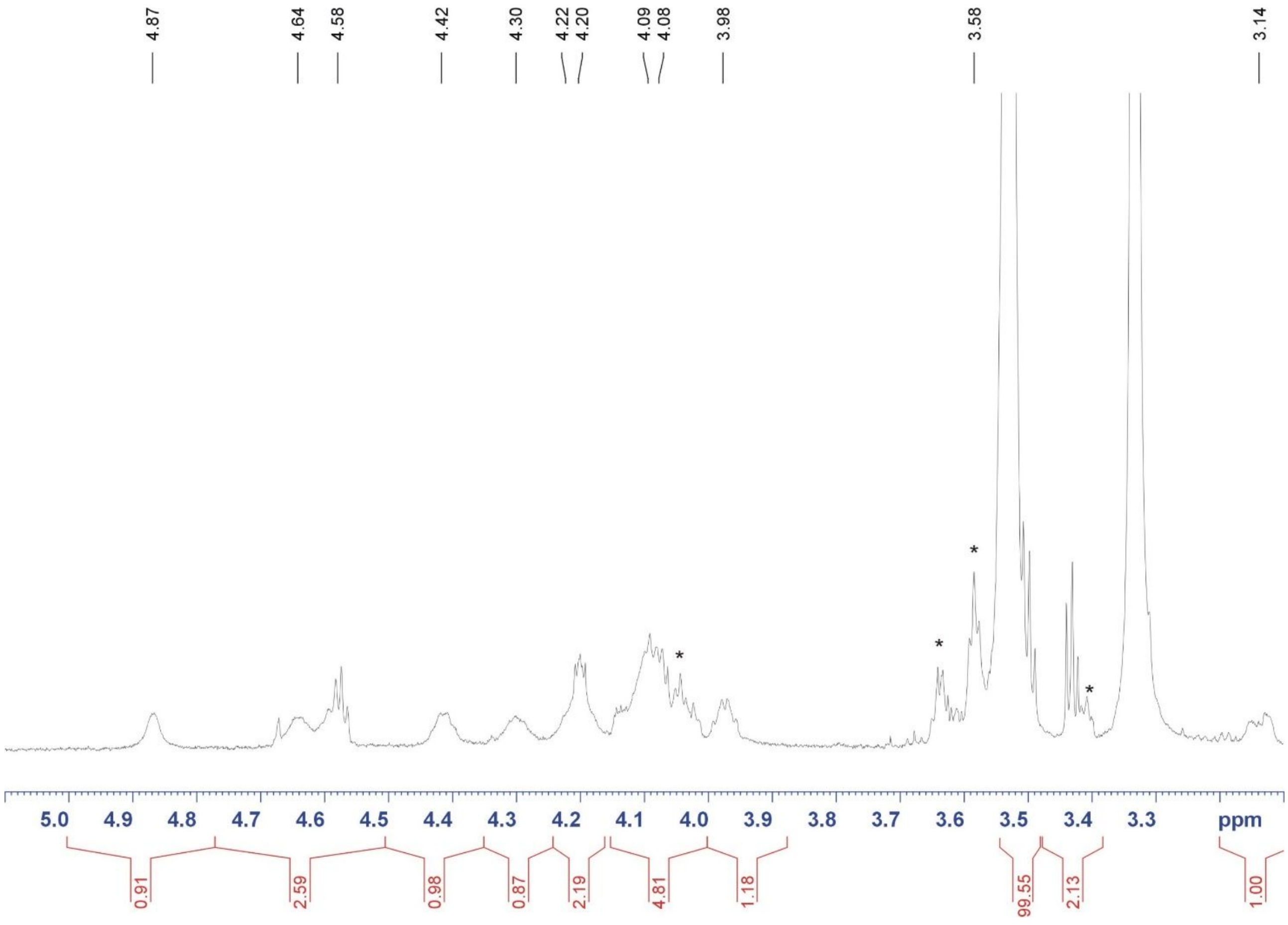

Figure 10S. Expansion of the 5.1-3.1 region

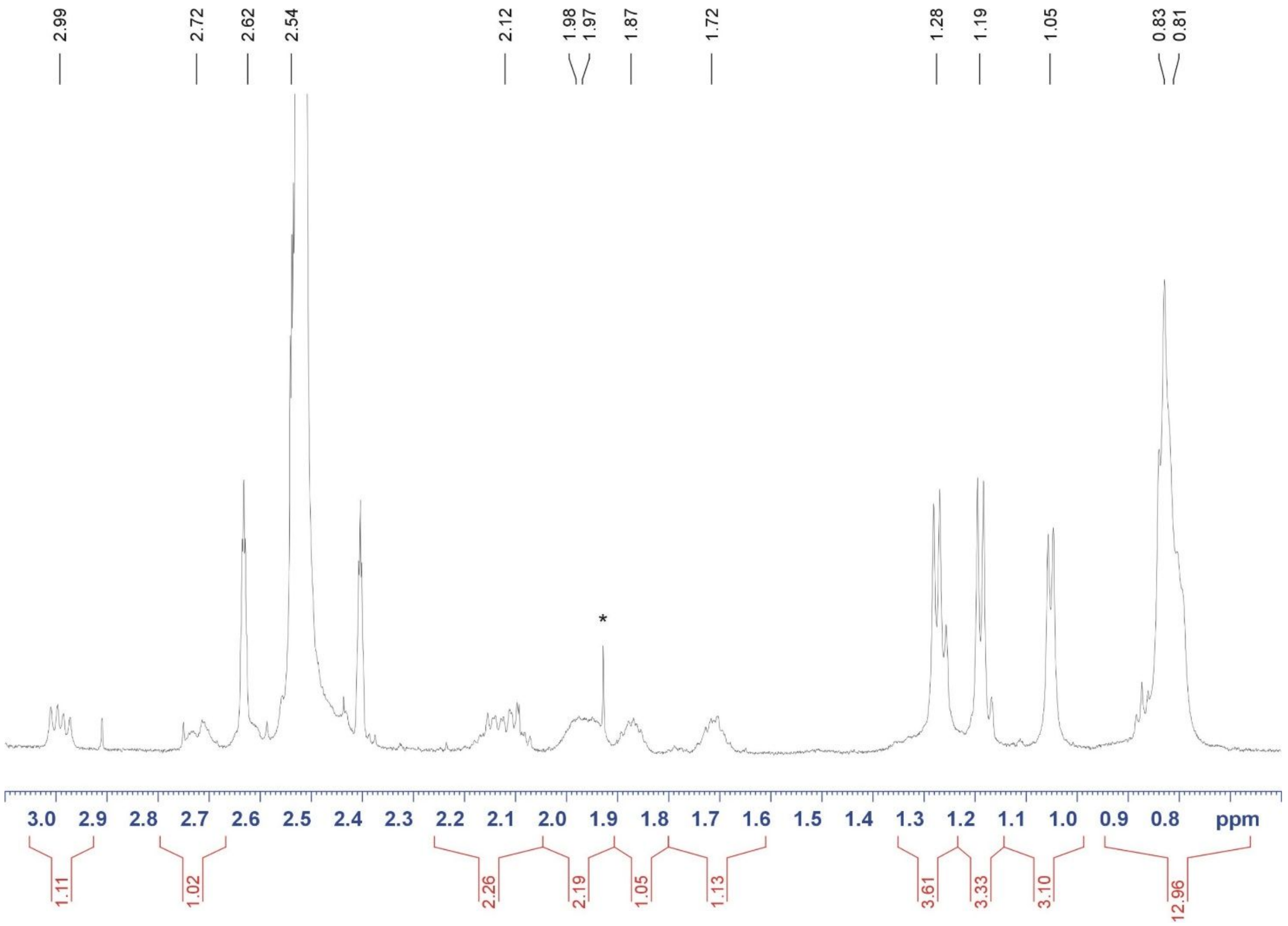

Figure 11S. Expansion of the 3.1-0.6 region

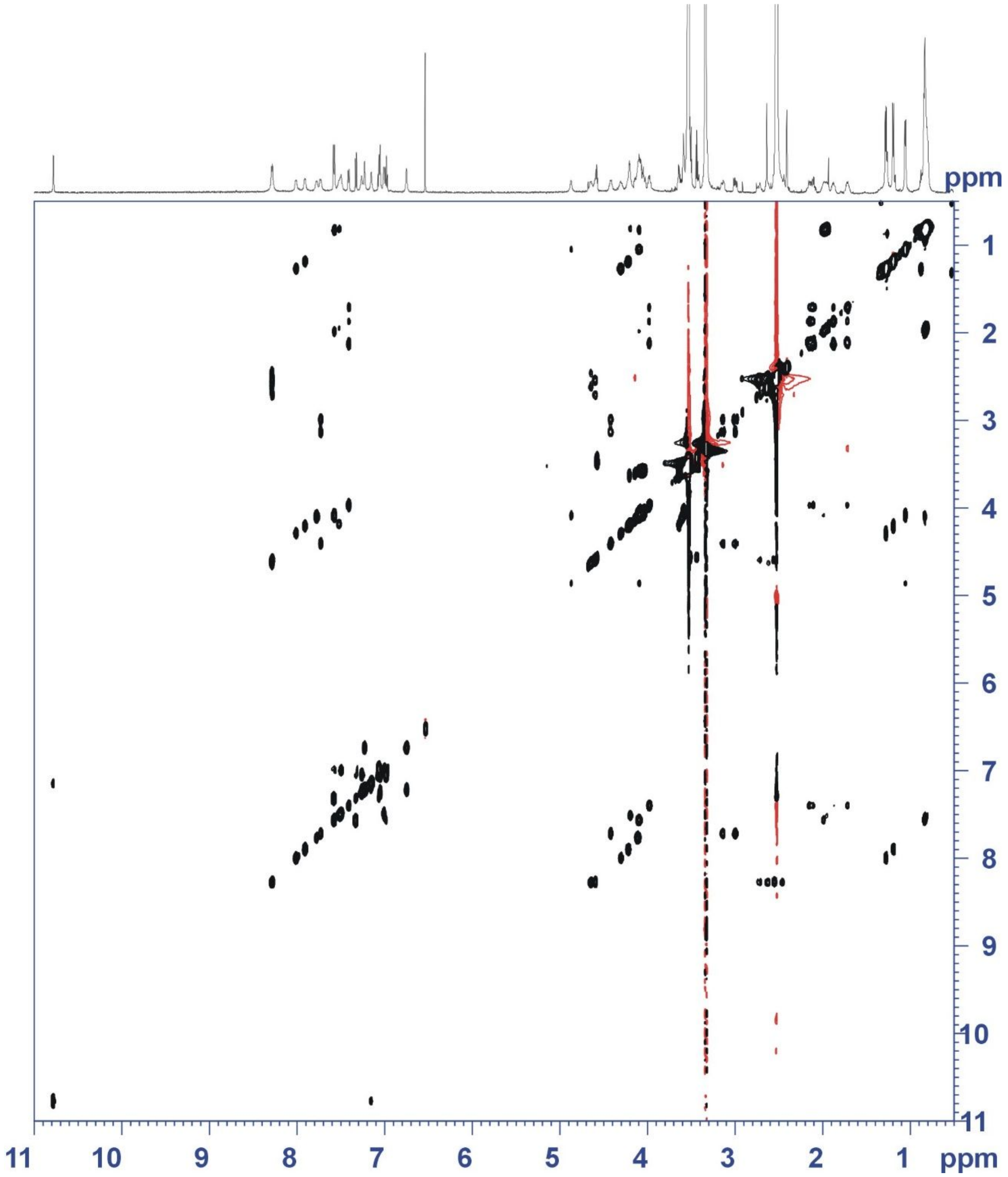

Figure 12S. Full view of the 2D-TOCSY NMR (mixing time 100 ms, 600 MHz, dmso-d_6, 298 K)

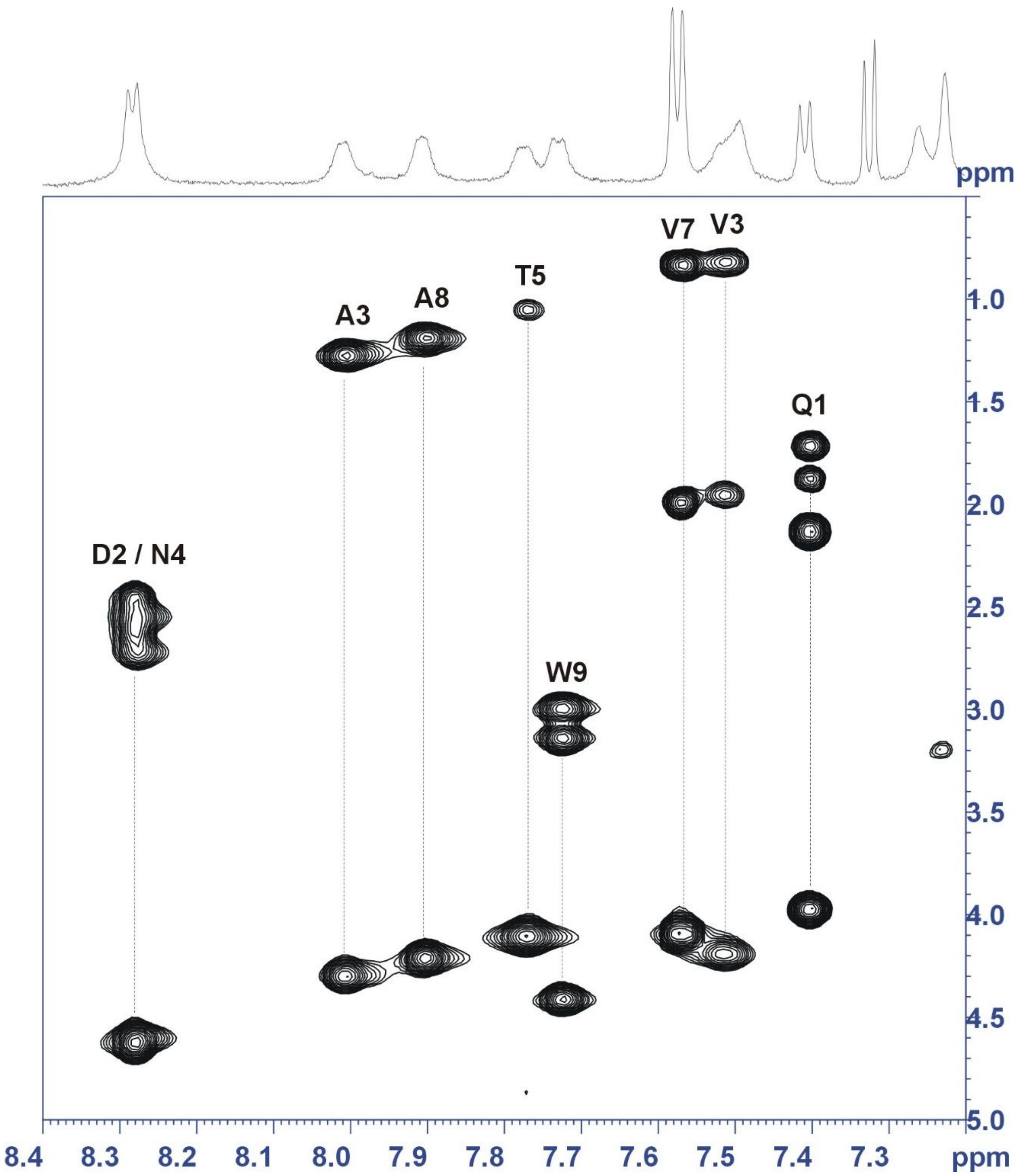

Figure 13S. Fingerprint region of the 2D-TOCSY NMR spectrum (600 MHz, dmso-d_6, 298 K) with assignment of the TOCSY stripes.

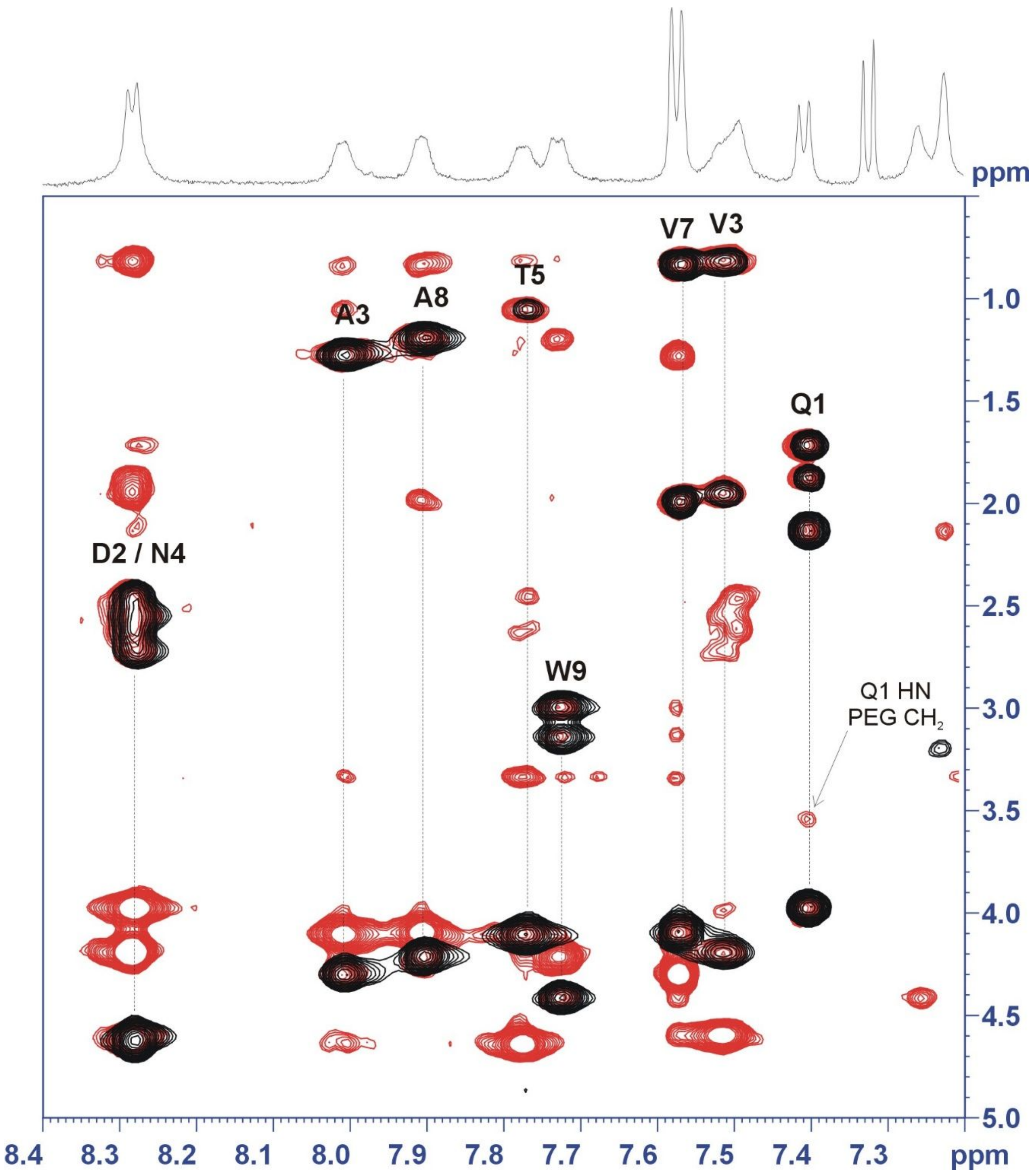

Figure 14S. Superposition of the fingerprint region of 2D-TOCSY (black) and 2D-NOESY (red) NMR spectra (600 MHz, dmso-d_6, 298 K). The NOESY peak between PEG methylene groups and Q^1 backbone carbamic H_N is shown.

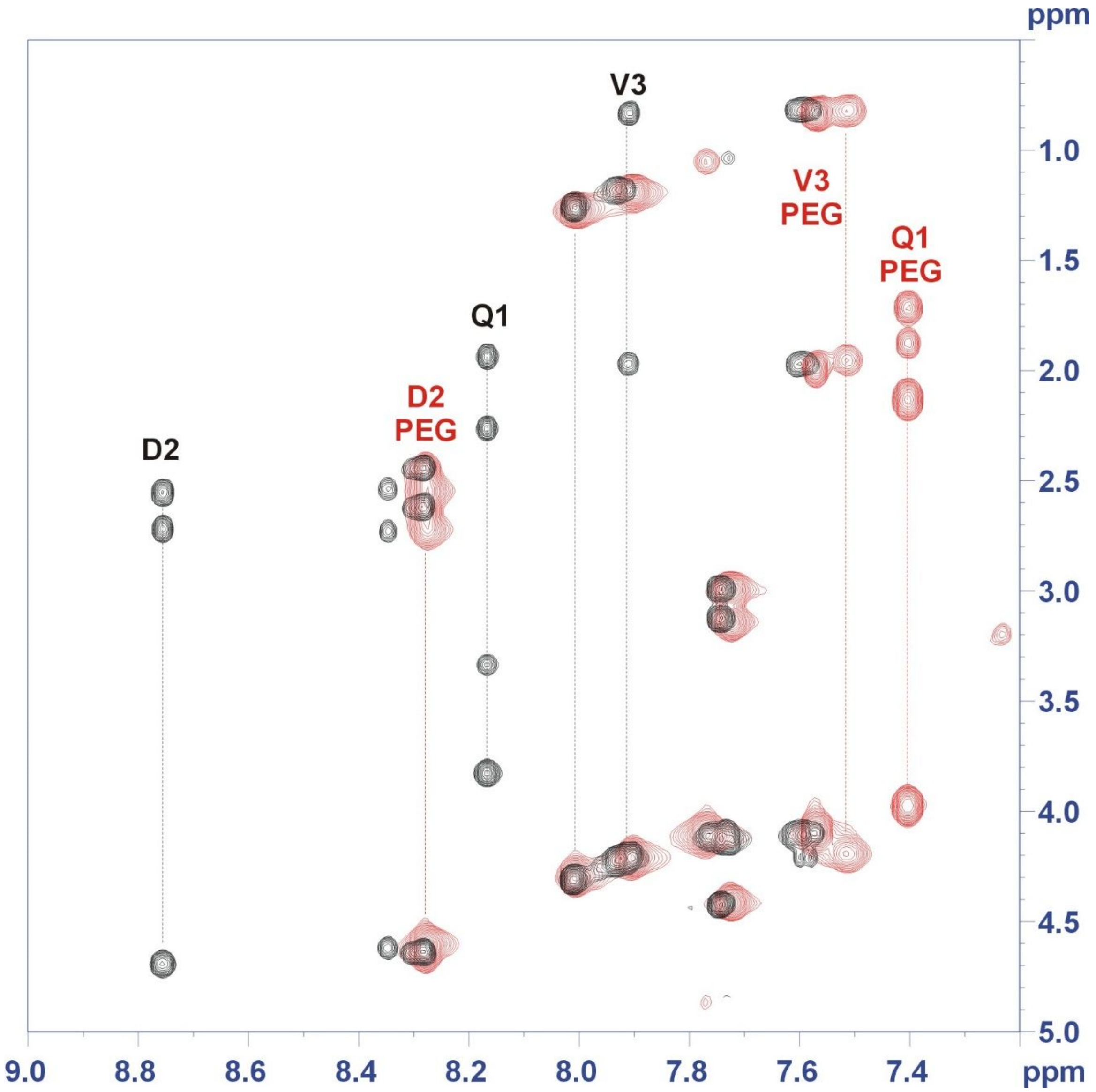

Figure 15S. Superposition of the 2D-TOCSY fingerprint region of A9-PEG (red) and A9-free (black). The functionalization of the N-terminus amino group mostly affects the amide resonance of Q^1, D^2 and V^3.

TIC analysis of A9–PEG–A9 degradation in cell-culture medium over 24 hours

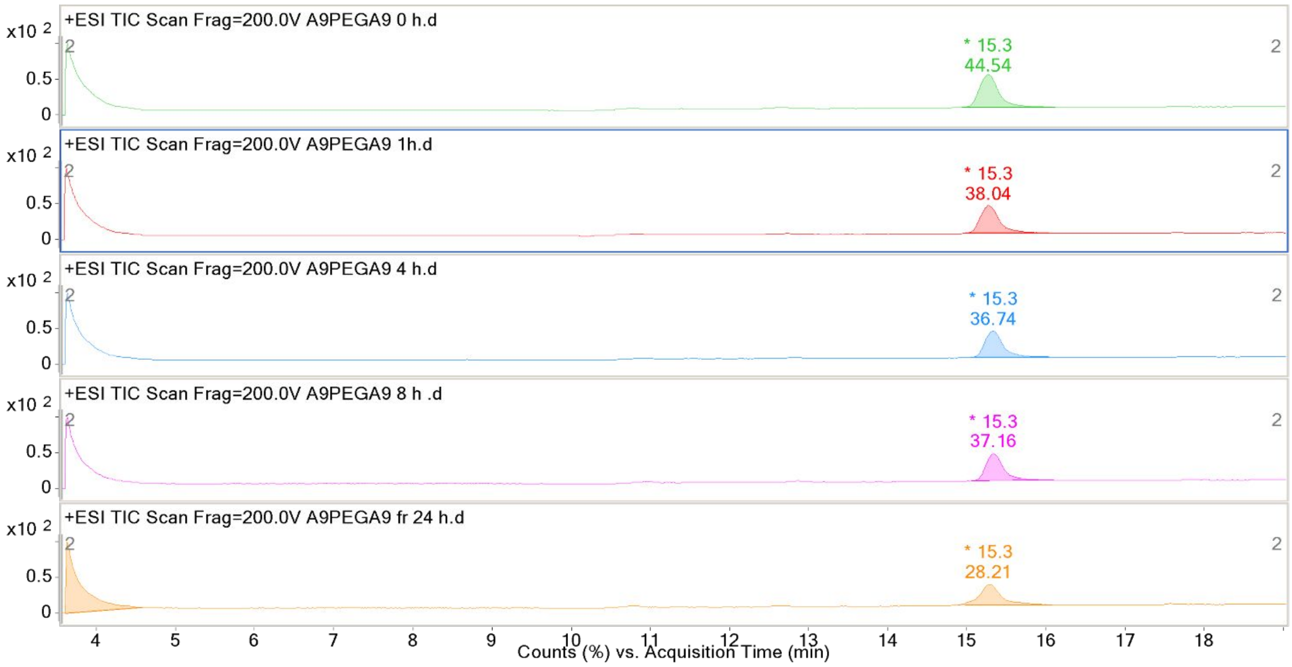

Figure 16S. TIC profile of A9–PEG–A9 at 0,1,4,8 and 24 hours

MONOMER A9-PEG-NHS

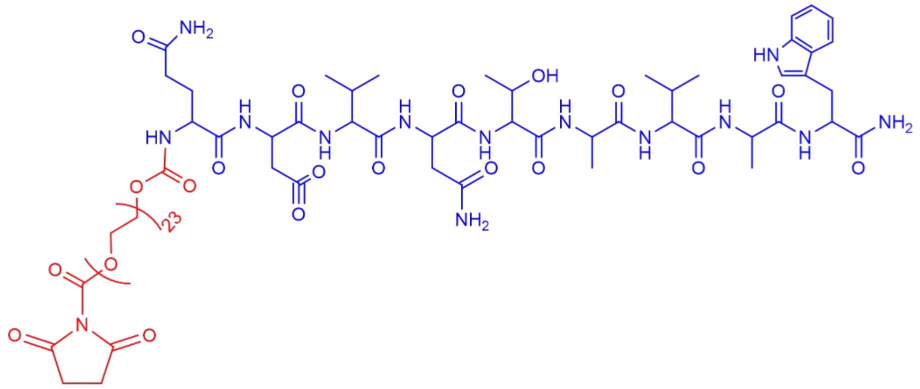

Figure 17S. Chemical structure of monomer A9-PEG-NHS

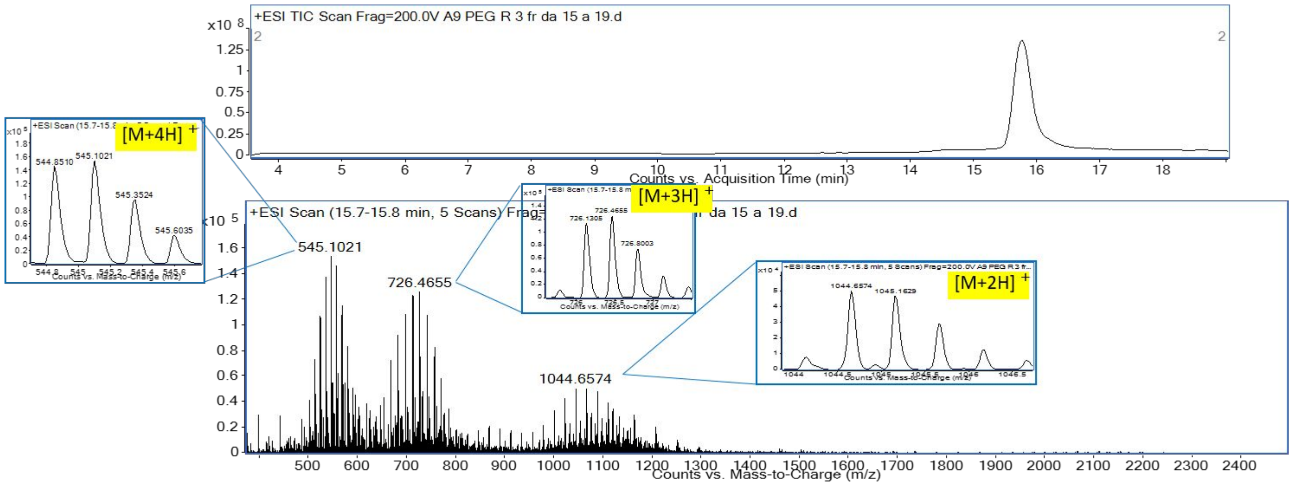

Figure 18S. TIC and MS spectra of monomer A9-PEG-NHS
